# Evaluation of Novel α-(Acyloxy)-α-(Quinolin-4-yl) Acetamides as Antiplasmodial Agents

**Published:** 2017

**Authors:** Ali Ramazani, Behnam Khosravani, Jafar Taran, Ali Ramazani

**Affiliations:** a *Zanjan Pharmaceutical Biotechnology Research Center, Zanjan University of Medical Sciences, Zanjan, Iran. *; b *Department of Chemistry, University of Zanjan, Zanjan, Iran.*

**Keywords:** *Plasmodium falciparum*, Quinolines, Cytotoxicity, HepG2, 3D7 strain

## Abstract

Because of expanding resistance to efficient and affordable antimalarial drugs like chloroquine, the search is continuing for more effective drugs against this disease. *In-vitro* antiplasmodial activity and cytotoxicity of α-(acyloxy)-α-(quinolin-4-yl) acetamides on *Plasmodium* *falciparum* and structure-activity relationships of this new class of Passerini adducts is described. The *in-vitro* antiplasmodial activity of compounds was tested against chloroquine sensitive 3D7 strain. Toxicity of active compounds was investigated on HepG2 cell line. Compounds 1, 20 and 22 showed significant antiplasmodial activity with IC_50_ value of 1.511, 1.373 and 1.325 µM, respectively. The active compounds did not show noticeable toxicity when tested against HepG2 cell line. The present results bring essential elements which will be used for the synthesis of more active derivatives of α-(acyloxy)-α-(quinolin-4-yl) acetamides.

## Introduction

According to the world health organization (WHO) report, there were about 219 million cases of malaria in 2010 and an estimated 660,000 deaths (1). Resistance is now common against all classes of antimalarial drugs and poses a growing problem in the malaria treatment, thus the biggest issue all over the world is to combat malaria with safe and effective medications and to avoid the emergence of drug-resistant malaria parasites (2). Of the various antimalarial drugs available, the aminoquinoline chloroquine was the agent of choice for many decades because of its safety, efficacy and affordability.

The quinoline scaffold is prevalent in a variety of pharmacologically active synthethic and natural compounds (3). The quinolines such as chloroquine, mefloquine, amodiaquine, primaquine, and quinine are historically among the most important antimalarial drugs ever used. The drugs from this group mostly act during the blood stages of the parasite’s life cycle but some target the hepatic stages as well (4). The quinolines are known to inhibit the polymerization of heme and prevent disposal of polymers from the food vacuole to the cytoplasm where hemozoin is formed. This leads to intraparasitic accumulation of free heme, which is highly toxic to the parasite (3).

Chloroquine (CQ), a 4-aminoquinoline, was first chemically synthesized in 1934, as a substitute for quinine. Since its discovery, CQ was the best antimalarial drug according to its safety, affordability, and efficacy. Despite this, the emergence and rapid spread of resistance of *Plasmodium falciparum* to CQ and other related antimalarials has dramatically reduced the therapeutic options (5) and have created an urgent need to discover new antimalarial agents (6). Researchers around the world have synthesized a large number of CQ analogues with the hope to overcome its drug-resistance properties (3, 7-10). In the context of our on-going research, we wished to synthesize novel compounds for screening against *P. falciparum* and to this end we reported synthesis of novel α-(acyloxy)-α-(quinolin-4-yl) acetamides by a three component reaction between an isocyanide, quinoline-4-carbaldehyde and arenecarboxilic acides (11).

## Experimental

*Chemicals*

All materials and reagents in this study were purchased from Merck (Darmstadt, Germany) and Sigma Aldrich (Steinheim, Germany). RPMI 1640 medium and AlbumaxI prepared from Gibco-Invitrogen (Paisley, Scotland, UK).

*Tested compounds*

**Figure 1:**
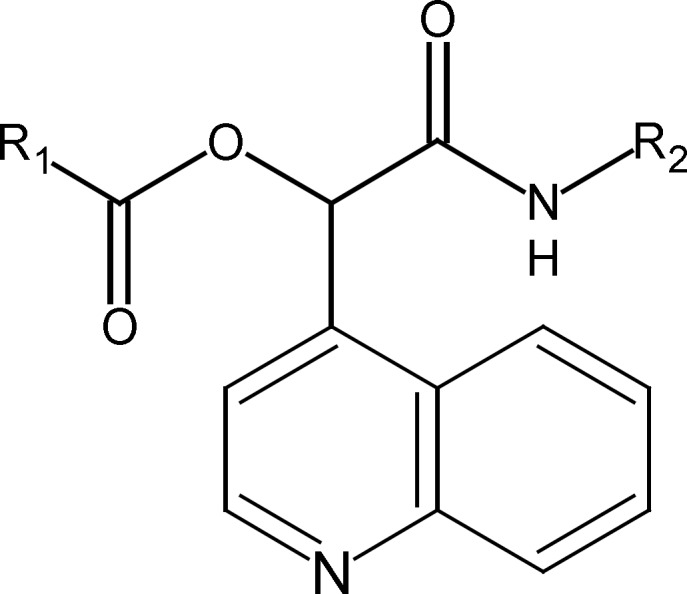
Structure of α -(acyloxy)-α-(quinolin-4-yl)acetamides.

We synthesized previously some novel α-(acyloxy)-α-(quinolin-4-yl) acetamides by a three component reaction between an isocyanide, quinoline-4-carbaldehyde and arenecarboxilic acides (11). The basic structure of α -(acyloxy)-α-(quinolin-4-yl) acetamides derivatives is indicated in Figure 1.

*Parasite Culture*

The *P. falciparum* 3D7 chloroquine-sensitive strain used throughout the study. *In-vitro* culture of *P. falciparum* was carried out according to the method described by previously (12-14). Briefly, parasites were cultured on human erythrocytes (blood group O^+^), provided by the Blood Transfusion Organization (Zanjan, Iran), in RPMI 1640 medium completed with 5% of human AB^+^ serum, 0.3 g/100 mL Albumax I, 25 mM HEPES, 19 mM sodium carbonate and 30 µg/mL gentamicin sulfate at pH 7.2. Type O^+^ erythrocytes were washed three times with RPMI 1640 and stored at 4 °C. 

Parasites were aerated in 25 mL flasks under 3% oxygen, 6% carbon dioxide and 91% nitrogen atmosphere. The medium was changed each day. 

*In-vitro antimalarial tests*

Pure compounds were dissolved in DMSO at concentration of 10 mg/mL and diluted with complete malaria culture medium to reach 1 mg/mL before use. Parasites were synchronized to the ring stage by sorbitol method described previously (15). From 2-fold dilution series (50-0.39 µg/mL) of compounds prepared in assay medium, 20 µL added to each well of 96-well microtiter plates in triplicate. One hundred eighty µL of synchronous *P. falciparum* culture (1% parasitemia, 2% hematocrit) added to each well reaching a final volume of 200 µL per well. Final DMSO concentration reached to 0.4% that was not toxic to parasite. Plates were incubated at 37 °C for 24 h. Chloroquine at 50% inhibitory concentration (0.7 µM) was used as positive control and parasitized erythrocytes without drug were used as negative control. After 24 h incubation, Giemsa stained thin smears were made and parasitemia was confirmed by the numeration of 1000 erythrocytes per slide. Data acquired by counting the erythrocytes in Giemsa stain were imported in Microsoft Excel spreadsheet and IC_50_ values were calculated from dose-response curves.

*In-vitro cytotoxicity assay*

In drug discovery approaches against malaria, one of the important strategies is the safety of active compounds against human. So, the toxicity of active compounds against *P. falciparum* was assessed on human hepatocellular carcinoma cell line (HepG2) by using MTT (3-[4,5-dimethylthiazol-2-yl] -2,5 diphenyl tetrazolium bromide) assay (16-17). The cells were cultured in RPMI 1640 medium enriched with 10% FBS (Fetal Bovine Serum) and incubated at 37 °C with 5% CO_2_ and 96% humidity. After several subcultures, cells were distributed in 96-well plates at 4,000 cells in 100 µL of culture medium and incubated for 24 h at the same condition to allow attachment of cells to the bottom of wells. Then culture medium removed and 100 µL of two-fold serially diluted concentrations of drug (400-1.56 µg/mL) added to each well in triplicate. Microtiter plates further incubated for 24 h in the same condition. Culture medium without drug was used as negative control. After the incubation time, the drug containing medium discharged and for evaluation of cell survival, 25 µL of MTT solution (4 mg/mL in PBS) added to each well and plates incubated for 3 h (in same condition). Then 100 µL of DMSO added to each well and plates were gently shaken to dissolve the formed formazan crystals. The absorbance of each well measured at 540 nm using an ELISA plate reader (Infinite M200, Tecan). The GI% (Growth Inhibition percent) was calculated using the formula Growth Inhibition% = 100 – (OD_test_ - OD_control_) × 100, where OD_test_ is the mean absorbance of treated cells and OD_control_ is the mean absorbance of a negative control. The cell survival of control assumed 100% and IC_50 _values generated from dose-response curves for each cell line. 

**Figure 2:**
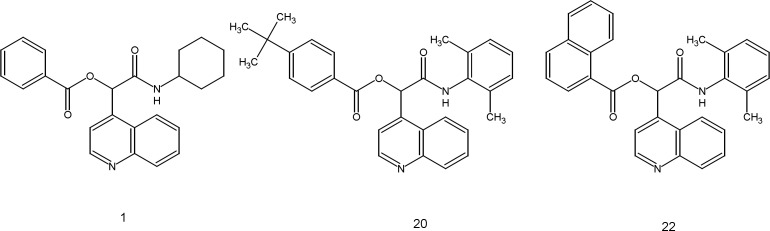
Structure of compounds 1, 20 and 22

**Table 1:** *In-vitro* antiplasmodial and cytotoxic activities of compounds 1-22**.**

**Compound**	**R**_1_	**R**_2_	***P. falciparum*** ** IC**_50_ ** (µM)**	**HepG2 IC**_50_ ** (uM)**	**SI**
1	Phenyl	cyclohexyl	1.511	229.93	152.17
2	4-tert-butylphenyl	cyclohexyl	2.635	190.6	72.33
3	4-methylphenyl	cyclohexyl	2.781	202.02	72.64
4	4-chlorophenyl	cyclohexyl	5.017	-	-
5	3-chlorophenyl	cyclohexyl	6.413	-	-
6	4-fluorophenyl	cyclohexyl	7.255	-	-
7	4-bromophenyl	cyclohexyl	5.792	-	-
8	4-iodophenyl	cyclohexyl	3.497	-	-
9	4-cyanophenyl	cyclohexyl	3.645	-	-
10	1-naphthyl	cyclohexyl	3.7	-	-
11	Phenyl	tert-butyl	4.346	-	-
12	4-tert-butylphenyl	tert-butyl	3.001	-	-
13	4-chlorophenyl	tert-butyl	5.849	-	-
14	3-chlorophenyl	tert-butyl	6.417	-	-
15	4-fluorophenyl	tert-butyl	5.056	-	-
16	4-bromophenyl	tert-butyl	5.103	-	-
17	4-cyanophenyl	tert-butyl	3.085	-	-
18	1-naphthyl	tert-butyl	6.953	-	-
19	Phenyl	2,6-dimethylphenyl	4.314	-	-
20	4-tert-butylphenyl	2,6-dimethylphenyl	1.373	199.07	144.98
21	4-chlorophenyl	2,6-dimethylphenyl	4.177	-	-
22	1-naphthyl	2,6-dimethylphenyl	1.325	254.27	191.90
CQ	-	-	0.7		

## Results and Discussion

The antimalarial activity of all compounds was evaluated against *P. falciparum* 3D7 chloroquine-sensitive strain. The antiplasmodial activities were determined as inhibitory concentrations at 50% parasite survival (IC_50_) in the strain and are tabulated in Table 1. Compounds 1, 20 and 22 showed significant antiplasmodial activity with IC_50_ value of 1.511, 1.373 and 1.325 µM, respectively. The IC_50_ values of these three compounds somewhat is near to CQ as the standard quinoline antimalaral drug. Compounds 2 and 3 also showed moderate activity with IC_50_ values of 2.635 and 2.781 µM. The rest of compounds did not show noticeable antiplasmodial activity. 

The cytotxicity of compounds with IC_50_ value less than 3 µM assessed on HepG2 cell line. Results of toxicity activity of the tested compounds and selectivity index (SI) are shown in Table 1. The SI is defined as the ratio of the HepG2 toxicity to the antiplasmodial activity and the higher selectivity should offer the potential of safer therapy without adverse effect in human. The 1, 20 and 22 compounds had high selectivity for *P. falciparum* than HepG2 cell line in comparison with other compounds.

Variation of different substituents has been explored to identify the better possible combination of substituents for the improvement of antimalarial potency. To study the effect of substituents on the antimalarial activity, variation of R1 substituents have been done while keeping the R_2_ substituent fixed. When cyclohexyl was placed at R_2_, compounds having 4-tert-butylphenyl(2),4-methylphenyl(3),4-iodophenyl(8),4-cyanophenyl(9)and 1-naphthyl (10) at R_1_ displayed almost equal antimalarial potency. Placement of 4-chlorophenyl(4),3-chlorophenyl(5),4-fluorophenyl(6)and 4-bromophenyl (7) significantly decreased the inhibitory activity as compared to the 2 and 3. When tert-butyl was placed at R2, mentioned substituents at R_1_,showed IC_50_ from 3 to 6.9 μM in compounds 11-18. Putting the 2, 6-dimethylphenyl at position R_2_, with phenyl (19) and 4-chlorophenyl (21) substituents at position R_1_ giving IC_50_ 4.314 and 4.177 μM, respectively. Among the 22 evaluated compounds of the series, compounds 1 (IC_50_ =1.511), 20 (IC_50_ =1.373) and 22 (IC_50_ =1.325) showed the better antimalarial potency. In this compounds R_1_ and R_2_ are: 1(phenyl, cyclohexyl), 20 (4-tert-butylphenyl, 2,6-dimethylphenyl),and22(1-naphthyl, 2,6-dimethylphenyl), respectively (Figure 2). These findings showed that compounds 1, 20 and 22 can be considered as new drug candidates for further evaluations in the next step of malaria drug discovery approaches.

## Conclusion

In conclusion, throughout the present study, we report the preliminary results regarding the structural requirements for the antiplasmodial activityofα-(acyloxy)-α-(quinolin-4-yl) acetamides. 

The present results bring essential elements, which will be used for the synthesis of more active derivatives of these compounds.
